# Human Neutrophil Defensins Disrupt Liver Interendothelial Junctions and Aggravate Sepsis

**DOI:** 10.1155/2022/7659282

**Published:** 2022-07-29

**Authors:** QiXing Chen, Yang Yang, YiHang Pan, LiHua Shen, Yan Zhang, Fei Zheng, Qiang Shu, XiangMing Fang

**Affiliations:** ^1^Department of Clinical Research Center, The Children's Hospital, Zhejiang University School of Medicine, National Clinical Research Center for Child Health, Hangzhou 310052, China; ^2^Department of Intensive Care Medicine, Sir Run Run Shaw Hospital, Zhejiang University School of Medicine, Hangzhou 310016, China; ^3^Department of Anesthesiology, The First Affiliated Hospital, Zhejiang University School of Medicine, Hangzhou 310003, China

## Abstract

Human neutrophil peptides 1-3 (HNP1-3), also known as human *α*-defensins, are the most abundant neutrophil granule proteins. The genes that encode HNP1-3, *DEFA1/DEFA3*, exhibit extensive copy number variations, which correlate well with their protein levels. Human and mouse studies have shown that increased copy numbers of *DEFA1/DEFA3* worsen sepsis outcomes. Additionally, high concentrations of HNP1-3 in body fluids have been reported in patients with sepsis. However, direct evidence for the pathogenic role of HNP1-3 proteins during sepsis progression is lacking. In current study, sepsis was induced by means of cecal puncture and ligation. Various doses of HNP-1 (low dose with 0.5 mg/kg body weight and high dose with 10 mg/kg body weight) or phosphate buffer saline were intraperitoneally administered to mice at six hours after sepsis onset. Survival rate was monitored, and vascular permeability, endothelial cell pyroptosis, and immunofluorescence of endothelial adherens junction protein vascular endothelial-cadherin were evaluated. The administration of a high dose of HNP-1 after sepsis onset led to increased mortality, more severe liver injury, and increased vascular permeability in the liver and mesentery. The injection of high dose of HNP-1 did not directly induce liver endothelial cell death but destroyed interendothelial junctions in the liver. Moreover, genetic deficiency of nucleotide-binding oligomerization domain-like receptor protein-3 or caspase-1 abrogated the high mortality and disrupted liver interendothelial junctions caused by high dose of HNP-1 during sepsis. This study directly demonstrates that neutrophil defensins play a key role in regulating endothelial stability during sepsis development.

## 1. Introduction

Defensins are short cationic peptides that constitute the front-line immune defense against various pathogens. Human neutrophil peptides 1-3 (HNP1-3), members of the *α*-defensin family, are the most abundant neutrophil-derived granule proteins, comprising approximately 5% of total neutrophil protein. HNP1-3 are arginine-rich and differ in sequence by one amino acid. They are released in response to neutrophil activation [1–3]. In addition to being potent antimicrobial agents, HNP1-3 act as immunomodulatory molecules with multiple functions, such as cytokine production induction and immune cell activation [2, 4, 5]. Moreover, in a very recent study, neutrophil *α*-defensin-1 was shown to promote clot formation [6].

HNP1-3 play important roles in the pathogenesis of sepsis [7–9]. The levels of HNP1-3 in the blood, bronchoalveolar lavage fluid, and sputum are greatly increased and rise up to 170 *μ*g/ml in some patients with sepsis [10, 11]. Our previous genetic association study found that *DEFA1/DEFA3* (the genes that encode HNP1-3) copy number variations are genetic risk factors for sepsis, and that individuals with high copy numbers of *DEFA1/DEFA3* are more susceptible to sepsis [12]. Using a transgenic mouse model, we further confirmed that mice with high copy numbers of *DEFA1/DEFA3* suffer from more severe pathophysiologic changes during sepsis progression [13]. The gene copy numbers of *DEFA1/DEFA3* correlate well with HNP1-3 protein levels [14]. A previous study showed that the neutrophil defensins HNP1-3 mediate the acute inflammatory response and lung dysfunction in a dose-related fashion [11]. However, direct evidence for how increased concentrations of neutrophil defensins act biologically during sepsis development is rather limited.

In present study, we aimed to determine whether administration of exogenous human neutrophil defensing HNP-1 affects sepsis progression and the mechanisms underlying these pathogenic changes in experimental septic mice.

## 2. Materials and Methods

### 2.1. Animal Model

Wild-type mice on a C57BL/6 genetic background were purchased from Shanghai Lab. Animal Research Center (Shanghai, China). Nucleotide-binding oligomerization domain-like receptor protein-3 (NLRP3) knockout (*Nlrp3*^−/−^) mice and caspase-1 knockout (*Casp1*^−/−^) mice on a C57/B6 background have been described previously [13, 15, 16].

The septic model was induced by performing cecal ligation and puncture (CLP) as previously described [13]. Six hours after sepsis onset, the mice were randomly assigned and given a low dose of HNP-1 (0.5 mg/kg, in 200 *μ*l phosphate-buffered saline (PBS); Chinese Peptide, Hangzhou, China) and a high dose of HNP-1 (10 mg/kg, in 200 *μ*l PBS) or PBS (200 *μ*l) intraperitoneally. The clinical score of mice was assessed at 24 hours and 48 hours after CLP. The symptoms of the maximum score of six are lethargy, piloerection, tremors, periorbital exudates, respiratory distress, and diarrhea. Each condition was scored as 1 [17]. The survival of the mice was monitored for 72 hours, and for general observation, some of the mice were sacrificed by decapitation at 48 hours after CLP performance. The experimental protocol was approved by the Animal Care and Use Committee of Zhejiang University (no. ZJU2015-105-01; Hangzhou, China), and the handling of animals was performed in accordance with the National Institutes of Health guidelines for ethical animal treatment.

### 2.2. Histopathology

For histopathology analysis, the lung and liver tissues were fixed in a 4% paraformaldehyde buffered solution for 24 hours and embedded in paraffin wax and cut into 4 *μ*m thick sections slices, followed by hematoxylin and eosin staining using standard procedures. Briefly, after rehydration, slides were placed in Mayer's hematoxylin solution for two minutes. Slides were rinsed with distilled water and acid alcohol prior to placement into 1% lithium carbonate. Slides were quickly dipped in 80% ethanol prior to exposure to eosin for 3 minutes. Finally, slides were dehydrated back through increasing concentrations of ethanol to xylene. Histological lung injury was quantitatively scored on a 5-point scale: 0, minimal damage; 1 to >2, mild damage; 2 to >3, moderate damage; 3 to >4, severe damage; and 4 +, maximal damage [18]. Liver injury was assessed for necrosis by standard morphologic criteria (loss of architecture, vacuolization, karyolysis, and increased eosinophilia), and the extent of necrosis was estimated by assigning a severity score on a scale from 0-4 as previously described: 0 = absent, 1 = mild, 2 = moderate, 3 = severe, and 4 = total necrotic destruction of the liver [19].

### 2.3. Bacterial Count Determination

To determine the bacterial load, the liver was harvested after perfusion and homogenized aseptically in cold PBS. Then, samples of the liver homogenates as well as peritoneal lavage fluid (PLF) and blood were serially diluted 10-fold and plated on Luria Bertani agar plates, which were incubated at 37°C for 12 to 16 hours under aerobic conditions. The numbers of bacterial colonies were calculated as colony-forming units, and data were log transformed for statistical analysis.

### 2.4. Cytokine Measurement

Plasma cytokine (interleukin (IL) -1*β*, IL-6, IL-10, and IL-18) levels were determined using commercially available enzyme-linked immunosorbent assay kits (Neobioscience, Shanghai, China; MULTISCIENCES, Hangzhou, China) according to the manufacturer's instructions.

### 2.5. Coagulation and Fibrinolysis Assay

Routine coagulation/fibrinolysis parameters in plasma, such as prolonged activated partial thromboplastin time (APTT), prothrombin time (PT), prothrombin time-international normalized ratio (PT-INR), thrombin time (TT), and fibrinogen, were measured on CA500 Series Automated Blood Coagulation Analyzer (Sysmex, Kobe, Japan) using commercially available assay kit (Siemens Healthcare Diagnostics Products GmbH, Marburg, Germany) according to the manufacturer's instructions.

### 2.6. Alanine Aminotransferase (ALT) and Aspartate Aminotransferase (AST) Assay

Plasma levels of ALT and AST were measured on cobas c 311 analyzer for clinical chemistry (Roche, Basel, Switzerland) using commercially available assay kit (Roche Diagnostics GmbH, Mannheim, Germany) according to the manufacturer's instructions.

### 2.7. In Vivo Permeability Assay

Vascular permeability was assessed with Evans blue dye (EBD) leakage from the blood into tissues *in vivo*. At 48 hours after CLP, mice were anesthetized, and EBD (20 mg/kg; Sigma-Aldrich, St. Louis, MO, USA) in a volume of 100 *μ*l was administered intravenously by tail vein injection. Thirty minutes after dye injection, the mice were transcardially perfused with normal saline through the left ventricle until blood was completely eliminated. Then, lung, liver, and mesentery tissues were harvested and dried at 55°C. Twenty-four hours later, the tissues were weighed and homogenized in 1 ml of formamide (Sigma-Aldrich, St. Louis, MO, USA) and then incubated at 55°C for 48 hours. Supernatants were collected after centrifugation at 5000 g for 30 minutes. The EBD in the supernatant was quantitated using a dual wavelength (620 and 740 nm) spectrophotometric method, correcting for contaminating heme pigments by using the formula OD_620_ (EBD) = OD_620_ − (1.426 × OD_740_ + 0.030) [20]. The extravasated EBD concentration in the tissue homogenate was calculated from a standard curve of EBD and expressed as the dye incorporated per mg of tissue.

### 2.8. Western Blotting

Liver tissues were homogenized in chilled lysis buffer (Thermo Fisher Scientific, Waltham, MA, USA), followed by incubation for 20 minutes on ice. After centrifugation at 12000 g for 10 minutes at 4°C, the supernatants were collected as protein samples. Protein content was determined using the Pierce™ BCA Protein Assay kit (Thermo Fisher Scientific, Waltham, MA, USA). Approximately 40 *μ*g of protein was loaded into a 12% *bis*-*tris* polyacrylamide gel (Thermo Fisher Scientific, Waltham, MA, USA) for separation. Afterward, the proteins were transferred to a polyvinyl difluoride membrane. After blocking, the membranes were blotted with primary antibodies against caspase-1 (Abcam, San Francisco, CA, USA), which was followed by blotting with a secondary antibody in a standard fashion. The membranes were developed using enhanced chemical luminescence (Biological Industries, Kibbutz Beit-Haemek, Israel) and exposed to X-ray films. *β*-Actin served as a protein loading control.

### 2.9. Dissociation of Liver Cells

After the mice were anesthetized and the abdominal cavity opened, the liver was removed and rinsed with Hank's Balanced Salt Solution. The sample was then digested using the Mouse Multi Tissue Dissociation Kit (Miltenyi Biotec, San Diego, CA, USA) on a gentleMACS dissociator (Miltenyi Biotec, San Diego, CA, USA). After digestion, the cell suspension was passed through a MACS SmartStrainer (70 *μ*m), and the cells were processed immediately for further applications.

### 2.10. Flow Cytometry Analysis of Cell Apoptosis and Pyroptosis

For apoptosis and pyroptosis analysis, single-cell suspensions from mouse liver tissue were incubated with allophycocyanin-conjugated anti-CD31 antibody (BD Biosciences, San Jose, CA, USA) for 30 minutes. After washing, the cells were incubated with FITC-conjugated Annexin V/PI from the Apoptosis Detection Kit II (BD Biosciences, San Jose, CA, USA), or carboxyfluorescein FLICA (ImmunoChemistry Technologies, Bloomington, MN) and PI following the manufacturer's instruction. The cells were then analyzed by flow cytometry. Acquisition was performed on 30,000 events using a BD LSR Fortessa (BD Biosciences, San Jose, CA, USA), and the data were analyzed by BD FACSDiva (BD Biosciences, San Jose, CA, USA).

### 2.11. Immunofluorescence

For immunofluorescence staining of the liver, the paraffin-embedded tissues were deparaffinized, rehydrated, and subjected to antigen retrieval by heating for 10 minutes in citrate buffer in a microwave oven. After blocking with 10% bovine serum for 1 hour at room temperature, the sections were incubated with primary antibody against mouse VE-cadherin (Abcam, Shanghai, China) at 4°C overnight, followed by exposure to Cy3-conjugated secondary antibody (Jackson ImmunoResearch Laboratories, West Grove, PA, USA). Nuclear staining was carried out with 4',6-diamidino-2-phenylindole using mounting medium for fluorescence (Vector Laboratories, Burlingame, CA, USA). All images were collected with a Nikon A1 confocal microscope (Nikon, Tokyo, Japan), and the fluorescence intensity was analyzed using Image J software (NIH, Bethesda, MD, USA). The fluorescence signals in at least five different high-power fields from each sample were quantified.

### 2.12. Statistical Analysis

Variables are expressed as the means ± SEMs. Differences among groups were analyzed using one-way analysis of variance (ANOVA) or Kruskal-Wallis test followed by Bonferroni or Dunn post hoc test as appropriate. Survival data were analyzed by the log-rank test. Statistical analysis was carried out using GraphPad Prism 7.0 (GraphPad Software Inc., La Jolla, CA, USA). A *P* value less than 0.05 was considered statistically significant.

## 3. Results

**Figure 1:**
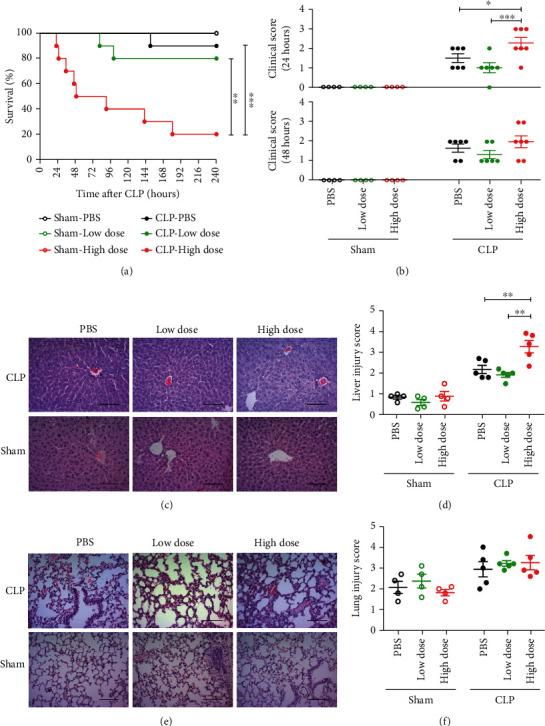
Administration of a high dose of HNP-1 aggravates sepsis outcome. Wild-type mice were intraperitoneally administered a high dose of HNP-1 (10 mg/kg body weight), a low dose of HNP-1 (0.5 mg/kg body weight), or PBS 6 hours after CLP or sham operation. (a) Survival over time. *n* = 10 mice in each CLP group, and *n* = 4 mice in each sham-operated group. (b) Clinical signs at 24 hours and 48 hours after CLP. The data shown are the means ± SEMs of 6-7 mice in each CLP group and 4 mice in each sham-operated group. (c) Representative images of hematoxylin and eosin-stained liver sections from mice 48 hours after CLP or sham operation. Scale bars, 50 *μ*m. (d) Quantification of the liver injury score based on the hematoxylin and eosin-stained liver sections in (c). The results shown are the means ± SEMs of 5 mice in each CLP group and 4 mice in each sham-operated group. (e) Representative images of hematoxylin and eosin-stained lung sections from mice at 48 hours after CLP or sham operation. Scale bars, 50 *μ*m. (f) Quantification of the lung injury score based on the hematoxylin and eosin-stained lung sections in (e). The results shown are the means ± SEMs of 5 mice in each CLP group and 4 mice in each sham-operated group. ^∗^*P* < 0.05, ^∗∗^*P* < 0.01, and ^∗∗∗^*P* < 0.001.

We first tested the effect of different doses of exogenous HNP-1 on the mortality and clinical symptoms of a model of CLP-induced sepsis. The intraperitoneal administration of HNP did not show any effects on the sham-operated mice (Figures 1(a) and 1(b)). Interestingly, the administration of a high dose of HNP-1 6 hours after sepsis onset led to the death of 80% of the mice during the 10-day observational period, while the low-dose- and PBS-administered groups had significantly lower mortality rates of 20% and 10%, respectively (Figure 1(a)). Also, the mice received high dose of HNP-1 after CLP showed more severe clinical symptoms of sepsis at 24 hours after sepsis onset as compared to those received low dose of HNP-1 and PBS, although these clinical features among the three groups were not significantly different at 48 hours after sepsis onset (Figure 1(b)).

We next observed the injury of vital organs such as liver and lung. Histopathologic analysis revealed that, compared with the other two groups, the mice given a high dose of HNP-1 showed more severe liver injury (Figures 1(c) and 1(d)) but no lung injury (Figures 1(e) and 1(f)) at 48 hours after CLP. Clinical chemistry analysis showed that the plasma levels of ALT and AST did not significantly increase in the mice received a high dose of HNP-1 (Supplementary Figures 1(a) and 1(b)).

**Figure 2:**
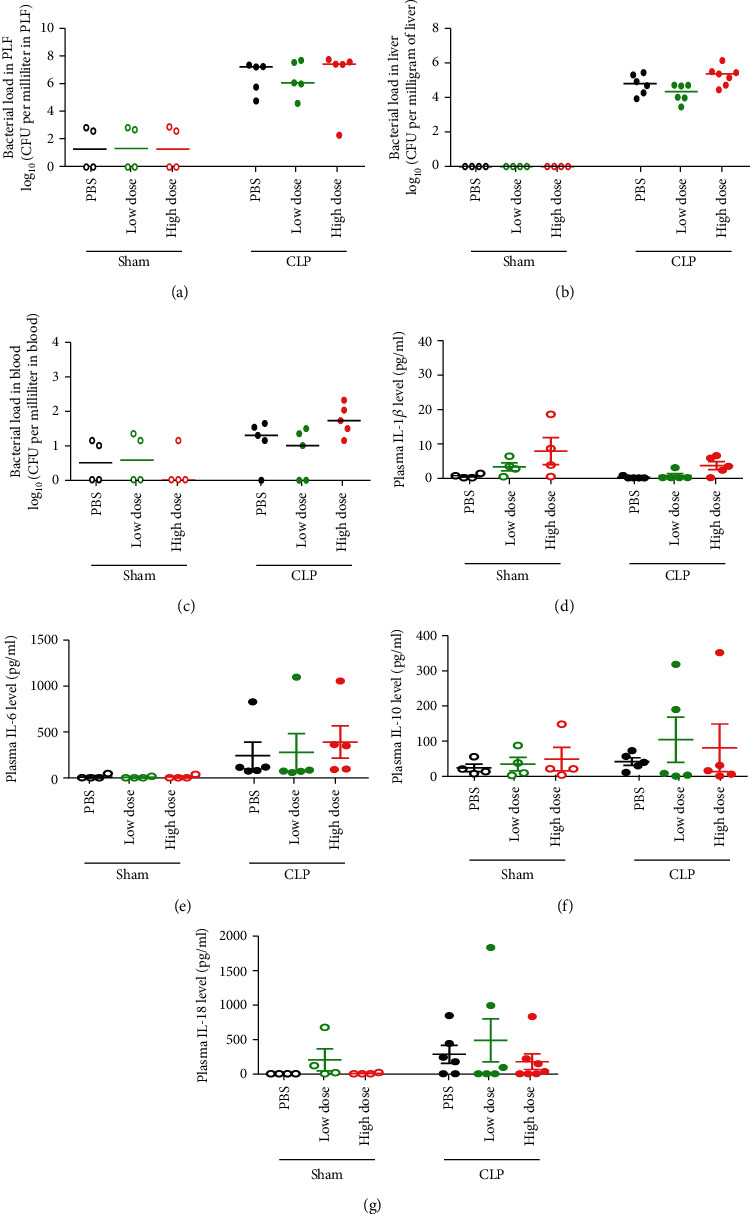
Characterization of bacterial load and inflammatory response during sepsis progression. Wild-type mice were intraperitoneally administered a high dose of HNP-1 (10 mg/kg body weight), a low dose of HNP-1 (0.5 mg/kg body weight), or PBS 6 hours after CLP or sham operation. (a–c) Bacterial load in the peritoneal lavage fluid (PLF; (a)), liver (b), and peripheral blood (c) at 48 hours after CLP or sham operation. The results shown are the medians of 5-7 mice in each CLP group and 4 mice in each sham-operated group. (d–g) Plasma levels of the inflammatory mediators IL-1*β* (d), IL-6 (e), IL-10 (f), and IL-18 (g) at 48 hours after CLP or sham operation. The results shown are the means ± SEMs of 5-7 mice in each CLP group and 4 mice in each sham-operated group.

At the onset of sepsis, endogenous bacteria from the cecum translocate into the blood compartment and then trigger systemic activation of the inflammatory response and subsequent organ dysfunction [21]. We found that both local (PLF and liver) and systemic (blood) bacterial burdens (Figures 2(a)–2(c)) as well as systemic inflammatory cytokines including IL-1*β*, IL-6, IL-10, and IL-18 (Figures 2(d)–2(g)) in all three groups were comparable.

**Figure 3:**
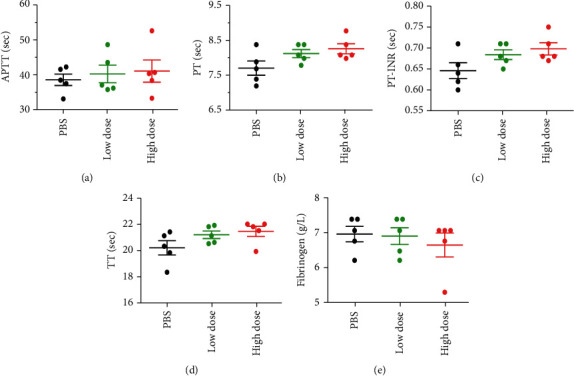
Effect of the administration of various doses of HNP-1 on the coagulation/fibrinolysis process during sepsis progression. Wild-type mice were intraperitoneally administered a high dose of HNP-1 (10 mg/kg body weight), a low dose of HNP-1 (0.5 mg/kg body weight), or PBS 6 hours after CLP. (a–e) Routine coagulation/fibrinolysis tests were performed 48 hours after CLP. (a) APTT. (b) PT. (c) PT-INR. (d) TT. (e) Fibrinogen. The results shown are the means ± SEMs of 5 mice in each group. APTT: prolonged activated partial thromboplastin time; PT: prothrombin time; PT-INR: prothrombin time-international normalized ratio; TT: thrombin time.

HNPs can inhibit fibrinolysis, hence, increase fibrin deposition and mediate the formation of thrombi [6, 22]. We further detected the coagulation/fibrinolysis status in the experimental mice. The administration of exogenous HNP-1 did not impact the routine coagulation/fibrinolysis process after sepsis challenge (Figures 3(a)–3(e)). Taken together, these findings suggest that the functions of HNP1-3, such as its antimicrobial activity, regulatory effect on inflammation, and thrombosis-promoting effects, did not play major roles during sepsis progression.

**Figure 4:**
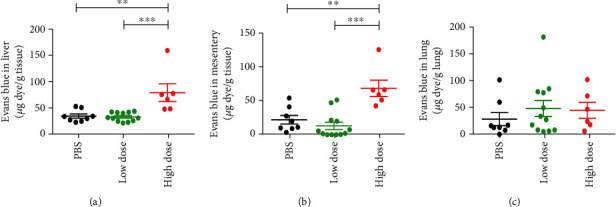
Administration of a high dose of HNP-1 impairs vascular permeability during sepsis progression. Wild-type mice were intraperitoneally administered a high dose of HNP-1 (10 mg/kg body weight), a low dose of HNP-1 (0.5 mg/kg body weight), or PBS 6 hours after CLP. (a–c) An *in vivo* Evans blue dye permeability assay in the liver (a), mesentery (b), and lung (c) at 48 hours after CLP. The results shown are the means ± SEMs of 6 mice in the high-dose group, 12 mice in the low-dose group, and 8 mice in the PBS group. ^∗∗^*P* < 0.01, and ^∗∗∗^*P* < 0.001.

**Figure 5:**
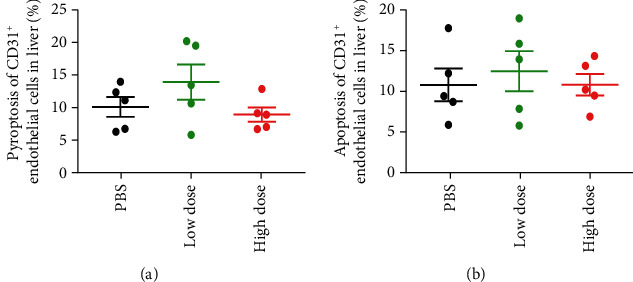
Effect of the administration of various doses of HNP-1 on liver endothelial cell death during sepsis progression. Wild-type mice were intraperitoneally administered a high dose of HNP-1 (10 mg/kg body weight), a low dose of HNP-1 (0.5 mg/kg body weight), or PBS 6 hours after CLP. (a) Quantification of pyroptotic endothelial cells in the liver at 48 hours after CLP using flow cytometry. The results shown are the means ± SEMs of 5 mice in each group. (b) Quantification of apoptotic endothelial cells in the liver at 48 hours after CLP using flow cytometry. The results shown are the means ± SEMs of 5 mice in each group.

In *DEFA1/DEFA3* transgenic mice, increased gene copy numbers of *DEFA1/DEFA3* worsen sepsis by inducing endothelial pyroptosis and vascular permeability in a canonical Nod-like receptor family pyrin domain containing 3 (NLRP3)/caspase-1 inflammasome dependent manner [13]. An Evans blue dye assay showed that administration of a high dose of HNP-1 led to increased vascular permeability in the liver (Figure 4(a)) and mesentery (Figure 4(b)) but not in the lung (Figure 4(c)). Moreover, western blot analysis in the liver tissues found that administration of a high dose of HNP-1 after sepsis onset activated caspase-1 in all tested mice, while administration of a low dose of HNP-1 after sepsis onset only activated caspase-1 in part of mice. There was no activated caspase-1 detected both in PBS-administered septic mice and sham-operated mice (Supplementary Figure 2). However, flow cytometry analysis showed that there was neither pyroptotic death nor apoptotic death in liver endothelial cells (Figures 5(a) and 5(b)), indicating that the intraperitoneal administration of a high dose of HNP-1 after sepsis onset may not directly lead to endothelial cell death in the liver.

**Figure 6:**
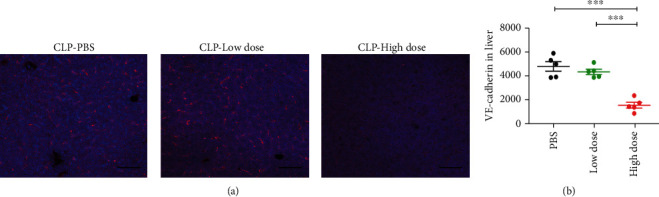
Administration of high dose of HNP-1 destroys liver endothelial cell junctions during sepsis progression. Wild-type mice were intraperitoneally administered a high dose of HNP-1 (10 mg/kg body weight), a low dose of HNP-1 (0.5 mg/kg body weight), or PBS 6 hours after CLP. (a) Representative images and (b) comparisons of VE-cadherin abundance in the liver at 48 hours after CLP are shown. The results presented are the means ± SEMs of 5 mice in each group. Scale bars, 50 *μ*m. ^∗∗∗^*P* < 0.001.

The vascular barrier consists of endothelial cells, cell-cell junctions, and extracellular components. Vascular endothelial (VE)-cadherin is the major component of adherens junctions, tightly regulated protein complexes that join adjacent endothelial cells and prevent vascular leakage [23, 24]. We then evaluated the abundance of VE-cadherin in the liver. Strikingly, compared to a low dose of HNP-1 and PBS, the administration of a high dose of exogenous HNP-1 after sepsis onset led to a significantly decreased level of VE-cadherin in the liver (Figures 6(a) and 6(b)).

**Figure 7:**
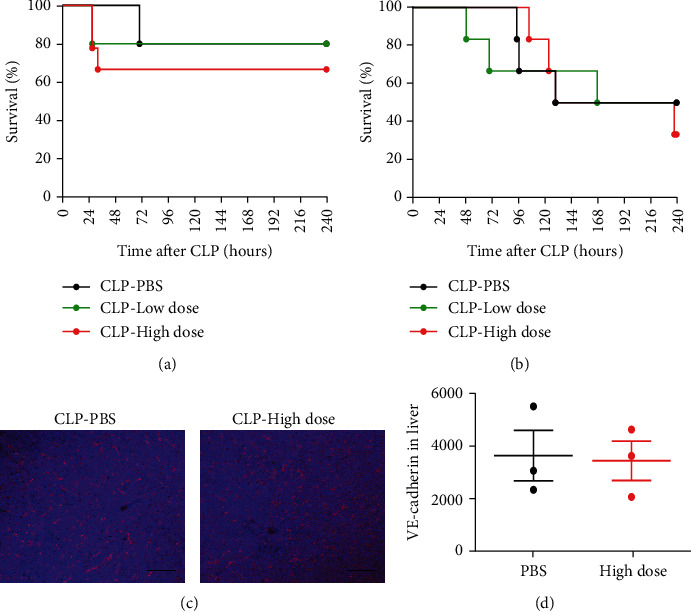
Genetic deficiency of NLRP3 or caspase-1 abrogated the damaging effects caused by high dose of HNP-1 during sepsis progression. *Nlrp3^−/−^* mice and *Casp1^−/−^* mice were intraperitoneally administered a high dose of HNP-1 (10 mg/kg body weight), a low dose of HNP-1 (0.5 mg/kg body weight), or PBS 6 hours after CLP. (a, b) Survival of *Nlrp3^−/−^* mice (a) and *Casp1^−/−^* mice (b) over time was monitored. In (a), *n* = 9 mice for the high-dose group, and *n* = 5 mice for the low-dose group and for the PBS group. In (b), *n* = 6 mice for each group. (c, d) Representative images (c) and comparisons of VE-cadherin abundance (d) in the liver of *Casp1^−/−^* mice administered a high dose of HNP-1 or PBS at 48 hours after CLP is shown. The results presented are the means ± SEMs of 3 mice in each group. Scale bars, 50 *μ*m.

Furthermore, we observed that when exogenous HNP-1 was intraperitoneally administered into *Nlrp3*^−/−^ and *Casp1*^−/−^ mice 6 hours after sepsis, the survival of mice administered a high dose of HNP-1 was comparable to that of mice administered a low dose of HNP-1 or PBS (Figures 7(a) and 7(b)). Also, the abundance of VE-cadherin in the livers of *Casp1*^−/−^ mice administered a high dose of HNP-1 was comparable to that in the PBS administered group (Figures 7(c) and 7(d)). Additionally, we found that the plasma and PLF levels of IL-1*β* and IL-18 in *Casp1*^−/−^ mice administered a high dose of HNP-1 after sepsis onset were not significantly decreased when compared to those in wild-type mice receiving a high dose of HNP-1 (Supplementary Figures 3(a)-3(d)).

## 4. Discussion

In current study, we found that high dose of human neutrophil defensins impaired vascular endothelial barrier and deteriorated liver injury and fatal outcome during sepsis development. These damages were not attributed to direct endothelial cell death induced by HNP-1 but attributed to destroyed interendothelial junctions in the liver. Furthermore, genetic deficiency of NLRP3 or caspase-1 abrogated the high mortality and disrupted liver interendothelial junctions caused by high dose of HNP-1 during sepsis.

Accumulated evidence suggests that HNP 1-3 act as a multifunctional mediator in host response to infection and sepsis. Since mice naturally lack neutrophil defensins, investigation of the role of human neutrophil defensins in sepsis and other diseases was largely dependent on genetic association studies. Recently, by employing transgenic mice, we found that mice carrying high copy number of *DEFA1/DEFA3* genes experienced more severe pathophysiological process after septic challenge [13]. In present study, through direct injection of exogenous human neutrophil defensin into septic mice, we further confirmed that high level of HNP-1 worsens the fatal outcome of sepsis. Combined these findings, a pathogenic role of high dose of HNP1-3 proteins in sepsis development is demonstrated.

During infection and inflammation, the levels of *α*-defensins in body fluid were elevated. Patients with meningitis infection and sepsis had extremely high levels of defensin in the plasma, ranging from 120 to 170,000 ng/ml [25]. It was speculated that the local concentrations of defensins in infected tissues are probably much higher [6]. An increased level of sputum defensins ranging from 300 to >1,600 *μ*g/ml in patients with cystic fibrosis has been reported [26]. In a previous study, Zhang et al. investigated the role of human neutrophil defensins in inflammatory lung disease through intratracheal instillation of exogenous human neutrophil defensins ranging from 5-30 mg/kg body weight of mouse (roughly correspond to a defensin concentration ranging from 1000 to 10000 *μ*g/ml in the lungs of the mouse) [11]. In current study, we used a high dose of human neutrophil defensin (10 mg/kg body weight of mouse in 200 *μ*l PBS, corresponding to a defensin concentration of 1250 *μ*g/ml in the peritoneal cavity of the mouse with a body weight of 25 g) for experiment. Although available data concerning the concentration of human neutrophil defensins in peritoneal lavage fluid from patients with abdominal infection and sepsis is rather limited, the intraperitoneal concentration of defensin used in present study was comparable and relevant to the concentration of defensins reported in other local infected tissues during sepsis and infectious diseases [10, 25].

The primary route of absorption after intraperitoneal injection is through the mesenteric vessels, which drain into the portal vein and pass through the liver [27, 28]. After intraperitoneal injection, HNP-1 slowly diffused from the peritoneal cavity into the venous circulation, reached the highest concentration in the portal vein and liver, and was then eliminated from the peripheral blood. Thus, it is not surprising that there was liver injury but no other vital organ injury occurred upon the administration of a high dose of HNP-1 after sepsis onset.

The liver microcirculatory bed is comprised of hepatic sinusoids, which are lined with a thin endothelium. Liver sinusoidal endothelial cells exert a key role in host defense and blood flow regulation and are a major target for injury during the early phases of sepsis. Junction proteins are essential for regulating the integrity of the endothelial monolayer and play a dominant role in the stability of endothelial cell contacts. The disruption of junction proteins occurs in the early-stage of enhanced vascular endothelial permeability and is one of the initial pathologic processes leading to endothelial dysfunction [23]. The intraperitoneal administration of a high dose of HNP-1 in wild-type mice after sepsis onset caused a decreased abundance of VE-cadherin in the liver sinusoidal endothelial cells, while this phenomenon was not observed when a high dose of HNP-1 was intraperitoneally administered to *Casp1*^−/−^ mice. Recent studies have also shown that NLRP3 inflammasome activation mediates the degradation of cell junction proteins and promotes vascular instability and paracellular permeability [29–31]. Thus, the intraperitoneal administration of a high dose of HNP-1 induces liver endothelial NLRP3/caspase-1 inflammasome activation and subsequently destroys cell junctions, thereby increasing vascular permeability after sepsis onset.

Sepsis is a frequently fatal condition characterized by an uncontrolled host response to microbial infection. Although intraperitoneal injection of a high dose of HNP-1 after sepsis onset impaired vascular endothelial barrier in the liver, we did not observe a much higher bacterial load in the liver. Since sepsis induction in present study is very mild, the magnitude of infection is also mild. Thus, it would be explained by that the administered HNP-1 might not affect hepatic immune cell functions, and the latter had enough ability to clear invading bacteria. Also, we did not observe significant changes in IL-1*β* and IL-18 levels between wild-type and *Casp1*^−/−^ mice administered a high dose of HNP-1 after sepsis onset. Our previous study found that HNP-1 promotes pyroptosis and IL-1*β* release through different roles of NLRP3 inflammasome [32], the mechanism of which may also involved in the present findings. Further studies to classify the activities of HNP-1 in mediating cell pyroptosis and IL-1*β* and IL-18 release as well as cell junction protein degradation are needed.

In summary, the current study found that administration of high dose of human neutrophil defensins destroyed liver interendothelial junctions via NLRP3/caspase-1 inflammasome in septic mice and led to increased liver vascular permeability and increased mortality in septic mice.

## Data Availability

The data used to support the findings of this study are available from the corresponding author upon request.

## Supplementary Materials

Supplementary Materials Supplementary Figure 1: effect of HNP-1 administration on biochemical test of liver function. Supplementary Figure 2: characterization of caspase-1 activation in the liver. Supplementary Figure 3: characterization of IL-1*β* and IL-18 levels in wild-type and *Casp1*^−/−^ mice during sepsis progression.
