# Supplementary material for: Human Neutrophil Defensins Disrupt Liver Interendothelial Junctions and Aggravate Sepsis
Source: Mediators Inflamm. 2022 Jul 29;2022:7659282. doi: 10.1155/2022/7659282 (PMC9355784; doi:10.1155/2022/7659282)
Supplement: Supplementary Materials — Supplementary Figure 1: effect of HNP-1 administration on biochemical test of liver function. Supplementary Figure 2: characterization of caspase-1 activation in the liver. Supplementary Figure 3: characterization of IL-1β and IL-18 levels in wild-type and Casp1−/− mice during sepsis progression. [file 7659282.f1.zip › Supplementary Materials.docx]

**Supplementary Figure legends**

**Figure 1.** Effect of HNP-1 administration on biochemical test of liver function. Wild-type mice were intraperitoneally administered a high dose of HNP-1 (10 mg/kg body weight), a low dose of HNP-1 (0.5 mg/kg body weight) or PBS 6 hours after CLP or sham operation. Plasma levels of Alanine aminotransferase (ALT) and Aspartate aminotransferase (AST) were tested at 48 hours after sepsis onset. The data shown are the means ± SEMs of 6-7 mice in each CLP group and 4 mice in each sham-operated group.

**Figure 2.** Characterization of caspase-1 activation in the liver. Wild-type mice were intraperitoneally administered a high dose of HNP-1 (10 mg/kg body weight), a low dose of HNP-1 (0.5 mg/kg body weight) or PBS 6 hours after CLP or sham operation. Activation of caspase-1 in the liver was analysed using immunoblotting. Representative result from two independent experiments was shown. One lane represents one mouse sample.

**Figure 3.** Characterization of IL-1β and IL-18 levels in wilt-type (WT) and *Casp1*^-/-^mice during sepsis progression. WT mice and *Casp1*^-/-^mice were intraperitoneally administered a high dose of HNP-1 (10 mg/kg body weight) 6 hours after CLP performance. (a-d) Plasma and peritoneal lavage fluid (PLF) levels of IL-1β (a, c) and IL-18 (b, d) at 48 hours after sepsis onset. The results shown are the means ± SEMs of 6-7 mice in each group.
