# Supplementary figures and images for: Human Neutrophil Defensins Disrupt Liver Interendothelial Junctions and Aggravate Sepsis
Source: Mediators Inflamm. 2022 Jul 29;2022:7659282. doi: 10.1155/2022/7659282 (PMC9355784; doi:10.1155/2022/7659282)

Supplementary Figure 2

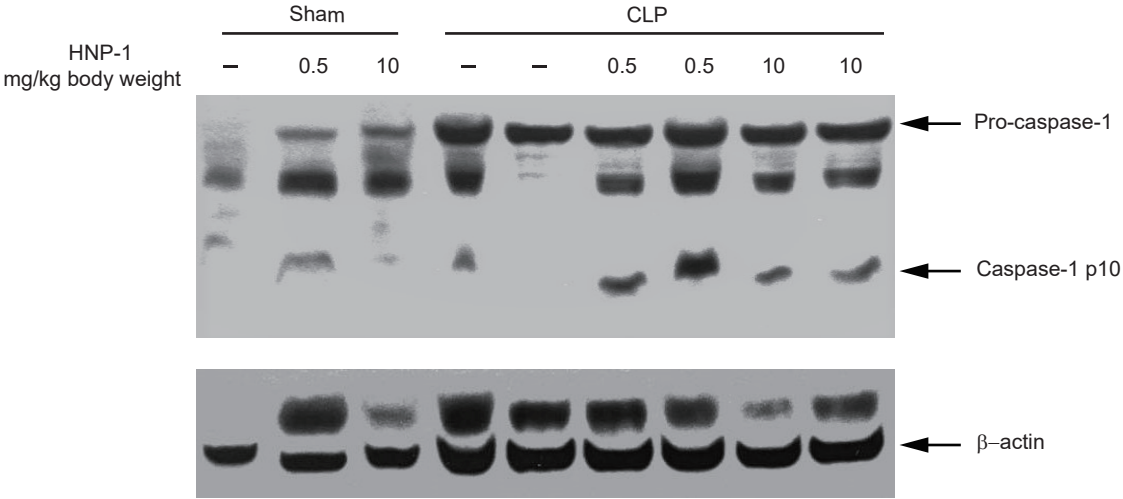

Supplement: Supplementary Materials — Supplementary Figure 1: effect of HNP-1 administration on biochemical test of liver function. Supplementary Figure 2: characterization of caspase-1 activation in the liver. Supplementary Figure 3: characterization of IL-1β and IL-18 levels in wild-type and Casp1−/− mice during sepsis progression. [file 7659282.f1.zip › Supplementary Figure 2.pdf]

### Supplementary Figure 3

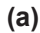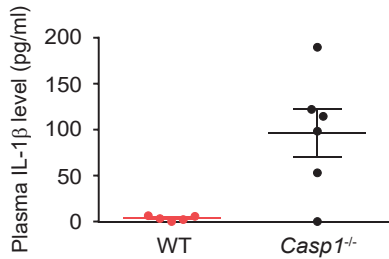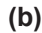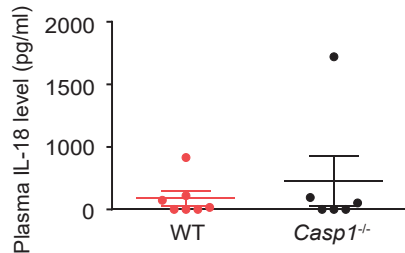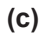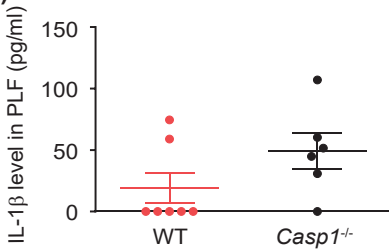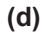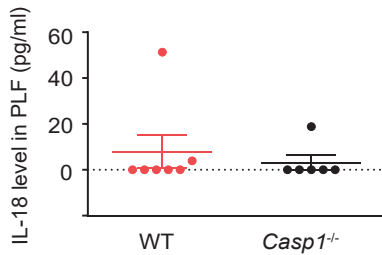

Supplement: Supplementary Materials — Supplementary Figure 1: effect of HNP-1 administration on biochemical test of liver function. Supplementary Figure 2: characterization of caspase-1 activation in the liver. Supplementary Figure 3: characterization of IL-1β and IL-18 levels in wild-type and Casp1−/− mice during sepsis progression. [file 7659282.f1.zip › Supplementary Figure 3.pdf]
